# Nanoparticles of a Pyrazolo-Pyridazine Derivative as Potential EGFR and CDK-2 Inhibitors: Design, Structure Determination, Anticancer Evaluation and In Silico Studies

**DOI:** 10.3390/molecules28217252

**Published:** 2023-10-25

**Authors:** Heba E. Hashem, Abd El-Galil E. Amr, Abdulrahman A. Almehizia, Ahmed M. Naglah, Benson M. Kariuki, Heba A. Eassa, Eman S. Nossier

**Affiliations:** 1 Department of Chemistry, Faculty of Women, Ain Shams University, Cairo 11757, Egypt; 2 Applied Organic Chemistry Department, National Research Center, Cairo 12622, Egypt; 3 Department of Pharmaceutical Chemistry, College of Pharmacy, King Saud University, P.O. Box 2457, Riyadh 11451, Saudi Arabia; 4 School of Chemistry, Cardiff University, Main Building, Park Place, Cardiff CF10 3AT, UK; 5 Department of Pharmaceutics and Pharmaceutical Technology, Faculty of Pharmacy (Girls), Al-Azhar University, Cairo 11754, Egypt; 6 Department of Pharmaceutical Sciences, School of Pharmacy and Physician Assistant Studies, University of Saint Joseph, West Hartford, CT 06117, USA; 7 Department of Pharmaceutical Medicinal Chemistry and Drug Design, Faculty of Pharmacy (Girls), Al-Azhar University, Cairo 11754, Egypt; 8 The National Committee of Drugs, Academy of Scientific Research and Technology, Cairo 11516, Egypt

**Keywords:** pyrazolo[3,4-*c*]pyridazine, nanoparticles, cytotoxicity, EGFR and CDK-2/cyclin A2 inhibition, docking simulation, ADME analysis

## Abstract

The strategic planning of this study is based upon using the nanoformulation method to prepare nanoparticles **4-SLNs** and **4-LPHNPs** of the previously prepared 4,5-diphenyl-1*H*-pyrazolo[3,4-*c*]pyridazin-3-amine (**4**) after confirming its structure with single crystal X-ray analysis. These nanoparticles exhibited promising cytotoxic activity against HepG-2, HCT-116 and MCF-7 cancer cell lines in comparison with the reference doxorubicin and the original derivative **4**. Moreover, their inhibitory assessment against EGFR and CDK-2/cyclin A2 displayed improved and more favorable impact than the parent **4** and the references. Detection of their influence upon cancer biomarkers revealed upregulation of Bax, p53 and caspase-3 levels and downregulation of Bcl-2 levels. The docking simulation demonstrated that the presence of the pyrazolo[3,4-*c*]pyridazin-3-amine scaffold is amenable to enclosure and binding well within EGFR and CDK-2 receptors through different hydrophilic interactions. The pharmacokinetic and physicochemical properties of target **4** were also assessed with ADME investigation, and the outcome indicated good drug-like characteristics.

## 1. Introduction

Cancer is one of the most serious health problems and the second highest cause of death worldwide. Conspicuously, the strategy for cancer treatment has remained basically unchanged, involving surgical removal of the malignancy followed by administration of either radiotherapy or chemotherapy, or both. Treatment failure may arise for a number of reasons, including resistance to chemotherapies, physiological hindrance against treatment, (such as cellular and blood–brain barriers that may obstruct transport of the drugs to targets), poor pharmacokinetics and systemic toxicity of the drugs [1,2,3].

Nanotechnology, which involves the use of particles with dimensions in the range of 1–100 nm, has shown promise in different medical applications, including cancer chemotherapeutics [4]. The basic concept of nanomedicine for anticancer drugs is the improvement of their therapeutic indices through optimization of their distribution in tissue and pharmacokinetics to speed up and facilitate their delivery to the site of action [5,6,7]. Nanomedicines with smaller systems (sub-100 nm) have displayed extravasation to a greater extent and penetration farther from the vasculature than larger systems, leading to improved efficacy. Additionally, systemic toxicity can be reduced or prevented through delivery of the drugs in nanoparticle formulations to the cancer cells via physiologically mediated active targeting and size-mediated passive targeting [8,9,10].

**Figure 1:**
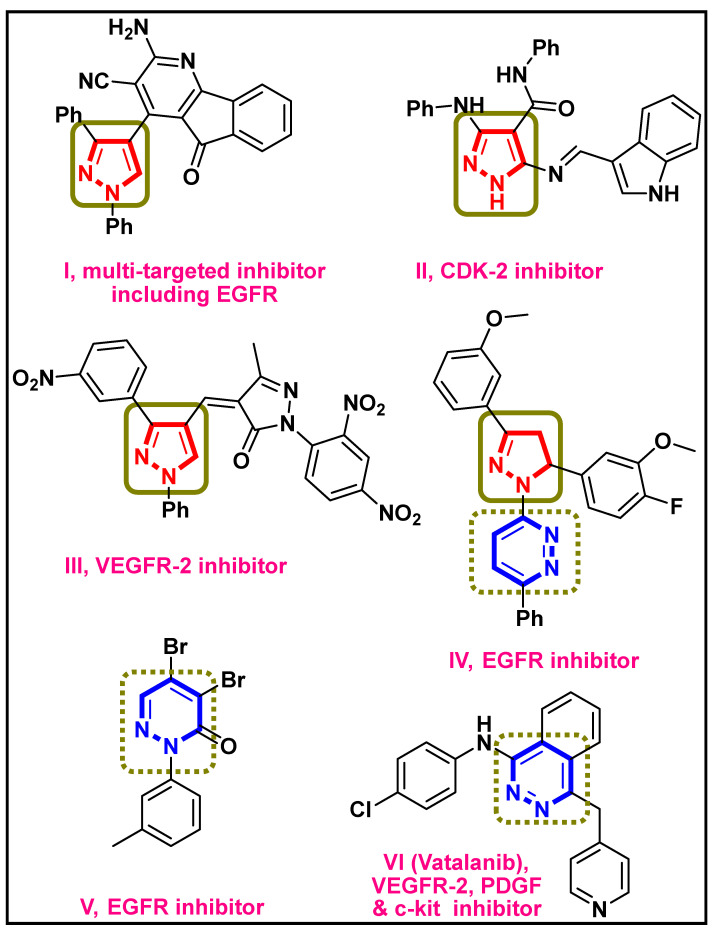
Some reported agents bearing pyrazole and pyridazine scaffolds with anticancer activity through different mechanisms.

Heterocyclic ring systems have been developed as dominant scaffolds in the design and discovery of new bioactive molecules. The pyrazole and pyridazine cores are established in a variety of biologically potent compounds having a broad spectrum of activities [11,12]. For example, the pyrazole target **I** (Figure 1), bearing 5-oxo-5*H*-indeno-[1,2-*b*]pyridine-3-carbonitrile, exerts its promising cytotoxic activity against human breast MCF-7, lung A-549 and colorectal HCT-116 cancer cell lines through the inhibition of six enzymes (AKT1, AKT2, BRAF V600E, EGFR, p38α and PDGFRβ) at 100 µM [13]. Pyrazole-indole hybrids **II** have demonstrated powerful antitumor activity against several human cancerous cell lines through excellent inhibition of CDK-2 kinase [14]. Conjugation of the pyrazolone moiety to the pyrazole core via CH linker in **III** synergized the anti-breast cancer activity with promising VEGFR-2 suppression [15]. Furthermore, the significant EGFR inhibitory activity of pyridazine-pyrazoline candidate **IV** explained its noticeable antiproliferative activity against breast T-47D and renal UO-31 cancer cell lines [16]. A new series of pyridazine derivatives, including **V**, have been described as effective anticancer agents that target EGFR [17]. An oral angiogenesis inhibitor called vatalanib **VI** is used to treat solid tumors in non-small cell lung cancer (NSCLC) and metastatic colon cancer. Vatalanib blocks VEGFR, PDGF and c-kit, three essential angiogenesis enzymes implicated in tumor growth and metastasis [18,19].

**Figure 2:**
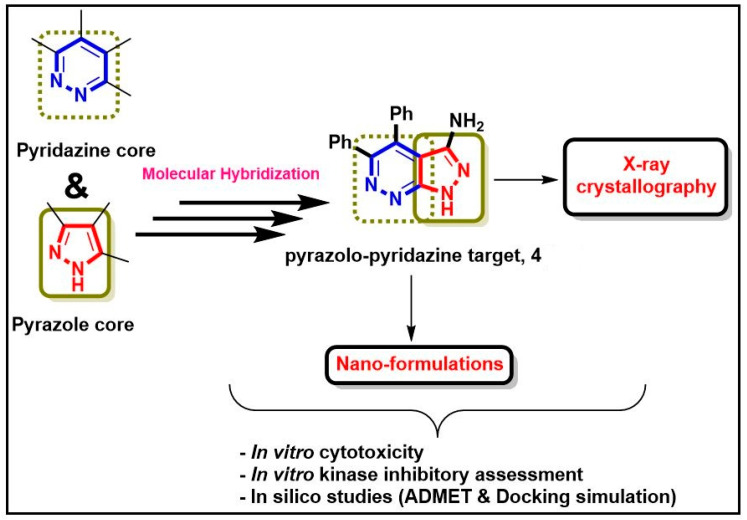
Rationalized design and further investigations of pyrazolo-pyridazine derivative 4 [20].

Inspired by the aforementioned knowledge, and our experience in cancer therapy, we have explored the generation of nanoparticles of the previously synthesized 4,5-diphenyl-1*H*-pyrazolo[3,4-c]pyridazin-3-amine (**4**) after confirming its structure with single crystal X-ray analysis (Figure 2). The nano-formulations (**4-SLNs** and **4-LPHNPs**) were evaluated for their cytotoxicity in comparison with the original derivative **4** against HepG-2, HCT-116 and MCF-7 cancer cell lines. Moreover, their kinase inhibitory activity against EGFR and CDK-2/cyclin A2 was assessed using erlotinib and roscovitine as standards, respectively. Various cellular parameters were also investigated, such as detection of Bax, Bcl-2, caspase-3 and p53 levels. Additionally, in silico studies including docking simulation against EGFR and CDK-2 and prediction of ADME were carried out.

## 2. Results and Discussion

### 2.1. Synthesis

**Scheme 1:**
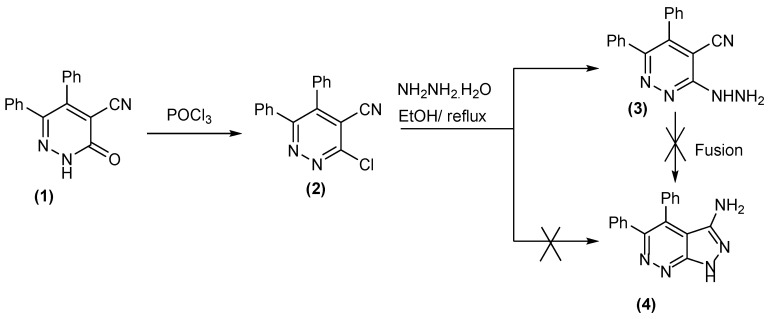
Synthesis and reaction of chloro-pyridazinone **2** with hydrazine hydrate to produce hydrazinyl-pyridazinone **3**.

In this work, 3-oxo-5,6-diphenyl-2,3-dihydropyridazine-4-carbonitrile (**1**) was synthesized, according to the literature [21], from the reaction of benzil and cyanoacetohydrazide under microwave irradiation. The reaction of **1** with phosphorous oxychloride afforded 3,4-diphenyl-5-cyano-6-chloro-pyridazine (**2**) in a good yield. When chloropyridazine derivative **2** was allowed to react with hydrazine hydrate in refluxing ethanol, it afforded the hydrazinyl derivative **3** instead of the expected cyclized fused pyrazolo-pyridazine derivative **4** as reported previously [20]. Additionally, the fusion of the hydrazinyl derivative **3** at its melting point did not give the predicted fused pyrazolo-pyridazine derivative **4** (Scheme 1).

**Scheme 2:**
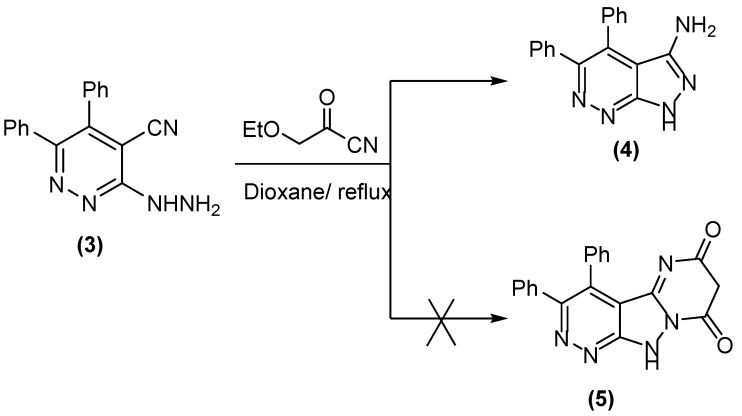
Synthesis of fused pyrazolo-pyridazine **4**.

**Figure 3:**
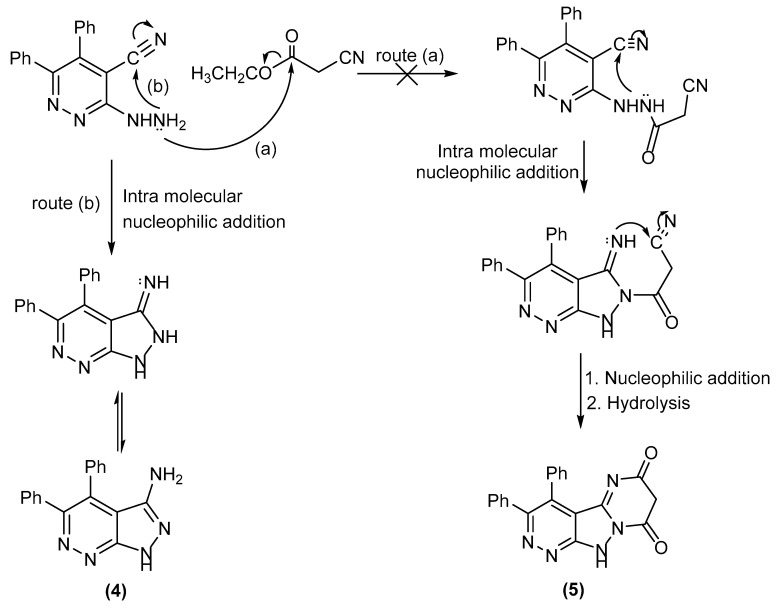
The suggested mechanistic steps for the formation of compounds **4** and **5**.

Treatment of hydrazinyl-pyridazinone **3** with ethyl cyanoacetate in refluxing dioxane gave pyrazolo-pyridazine derivative **4**, instead of the predicted polynuclear pyrimido-pyrazolo-pyridazine derivative **5** (Scheme 2), and the suggested mechanistic steps for the formation are illustrated in Figure 3.

The formation of compound **4** was confirmed with X-ray analysis, ^1^HNMR, ^13^NMR, IR and mass spectrum. Its IR spectrum is devoid of any signal for CN and CO groups. The ^1^HNMR spectrum is in agreement with the proposed structure, as it shows one broad singlet signal in the up-field region for NH_2_ protons, a broad singlet signal in the down-field region for the NH proton, as well as the aromatic protons (see Section 3).

### 2.2. Crystal Structure of Compound **4**

**Figure 4:**
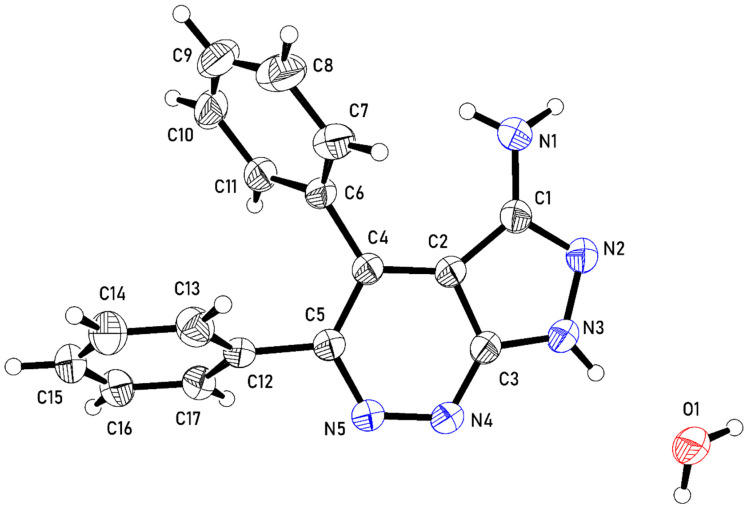
An Ortep representation of the asymmetric unit of the crystal structure of **4**, showing 50% probability atomic displacement parameters.

The crystal comprises molecules of **4** and water of crystallization (Figure 4). The molecular structure of **4** consists of a planar pyrazolopyridazinamine group (**A**, C1–C5, N1–N5) and two phenyl rings (**B**, C6–C11, and **C** C12–C17). Selected molecular geometry for **4** is shown in Supplementary Data (Table S1). In the crystal structure, the two phenyl rings of the molecule are twisted from the plane of the pyrazolopyridazinamine group with twist angles **A**/**B** and **A**/**C** of 62.46(4)° and 60.90(4)°, respectively. An N-H…O hydrogen bond links the molecule of **4** to the water of crystallization which in turn donates O-H…N bonds to two other molecules of **4** in the crystal (Supplementary Data, Table S1). N-H…N interactions between molecules of **4** also occur in the crystal structure.

### 2.3. Development of the Target **4** into Nano-Formulations (4-SLNs and 4-LPHNPs)

As previously reported, pyrazolo-pyridazine **4** displayed potential inhibitory activity against CDK1/cyclin B [20], glycogen synthase kinase-3 (GSK-3) and CDK2/cyclin A, implying that it plays an important role in the control of the cell cycle, transcription and insulin action [22]. There was also an attempt to synthesize a series of pyrazolo[3,4-*c*]pyridazine **4** by substituting the two phenyl rings at positions 4 and 5 with various groups in order to improve its effectiveness against the CDK family and explore the structure–activity relationship. It was observed that the derivatives containing electron-withdrawing groups in the para position of both aromatic rings were inactive against CDK1/cyclin B and all examined cell lines, including human cervical cancer (HeLa), prostate carcinoma (PC-3) and colon carcinoma (HT-29). The derivative containing an electron-donating group, on the other hand, showed inhibitory effects against CDK1/cyclin B, albeit with a 5 fold decrease in activity when compared to the parent **4** [20]. Despite the fact that the synthesized pyrazolo-pyridazine derivatives met the Lipinski rule criteria, they did not show cytotoxic effects on the cell lines tested. This finding could be explained by their difficulties in penetrating cell membranes [20]. As a result of the aforementioned discovery, we attempted to develop nano-formulations of pyrazolo-pyridazine.

#### Characterization of Developed Nano-Formulations (Particle Size and Zeta Potential)

**Figure 5:**
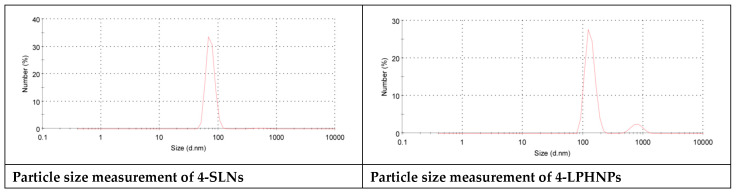
Particle size measurements of **4-SLNs** and **4-LPHNPs**.

The important parameters in the determination of the quality and stability of nanoparticles formulations include their particle sizes and zeta potentials. **4-SLNs** showed mean particle size diameter of 74.08 nm and zeta potential of −40.9 mV. Upon addition of polymer, **4-LPHNPs** afforded a mean particle size diameter of 133.1 nm and zeta potential of 21.6 mV (Figure 5). The **4-SLNs** displayed a small particle size in the nanosize range (74.08 nm) whereas **4-LPHNPs** exhibited a larger particle size (133.1 nm) but also in the nanosize range. The increase in the particle size can be attributed to the coating of SLNs with Eudragit L100 [23]. Regarding zeta potentials of the developed nanoparticles, **4-SLNs** were found to have a negative charge of −40.9 mV, which indicated high repulsive force and consequently long-term stability [24,25]. In the case of **4-LPHNPs**, the cationic nature of Eudragit L100 reduced the density of the electron cloud surrounding the nanoparticle surface and changed the potential into a positive charge of 21.6 mV. These results are consistent with Eudragit L100 being adsorbed on the nanoparticle surface and are associated with the affinity of the suppocire to Eudragit long chains [26].

### 2.4. Biological Evaluation

#### 2.4.1. In Vitro Cytotoxicity

**Figure 6:**
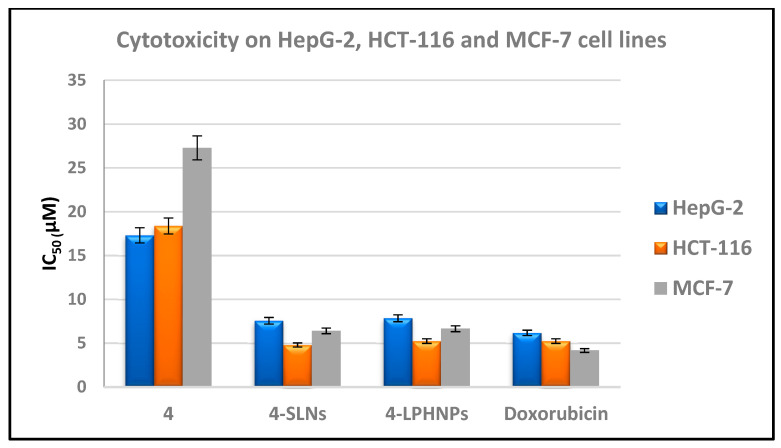
Cytotoxicity of targets **4**, **4-SLNs** and **4-LPHNPs** against HepG-2, HCT-116 and MCF-7 cell lines cell lines according to the MTT assay.

The antiproliferative activity of target **4** and its nanoparticles **4-SLNs** and **4-LPHNPs** was screened in vitro against human liver HepG-2, colorectal (HCT-116) and breast (MCF-7) cancer cell lines through the colorimetric MTT assay [27,28], and results were compared with the reference drug, doxorubicin. The IC_50_ values of the screened derivatives in μM were calculated and are shown in Table 1 and Figure 6. It is notable that target **4** displayed weak cytotoxic activity against MCF-7 and was moderate against cell lines HepG-2 and HCT-116 (IC_50_ = 27.29, 17.30 and 18.38 µM, respectively) compared to the standard (IC_50_ = 4.17, 6.18 and 5.23 µM, respectively). On the other hand, the nano-formulations (**4-SLNs** and **4-LPHNPs**) displayed potency similar or superior to the standard (IC_50_**_4-SLNs_** range 4.80–7.56 µM and IC_50_**_4-LPHNPs_** range 5.24–7.85 µM against the tested cell lines).

#### 2.4.2. In Vitro Kinase Inhibitory Assessment against EGFR and CDK-2

Due to the cytotoxic efficacy of compound **4** and its nanoparticles **4-SLNs** and **4-LPHNPs**, it was of benefit to study the mode of action through assessment of their inhibitory activity against EGFR and CDK-2/cyclin A2 [29,30,31,32,33]. Their IC_50_ values (μM) were calculated (Table 2) using erlotinib and roscovitine as standards, respectively.

Interestingly, the pyrazolo-pyridazine **4** displayed moderate inhibitory activity against EGFR and CDK-2/cyclin A2 (IC_50_ = 0.391 and 0.55 μM, respectively) compared with the references, erlotinib and roscovitine (IC_50_ = 0.126 and 0.32μM, respectively). The impact of the nano-formulations (**4-SLNs** and **4-LPHNPs**) was elevated against both enzymes to afford superior inhibitory activity in comparison with the standards (IC_50_**_4-SLNs_** = 0.088 and 0.18 µM and IC_50_**_4-LPHNPs_** = 0.096 and 0.22 µM against EGFR and CDK-2/cyclinA2, respectively).

#### 2.4.3. Effect of Target 4 and Its Nanoparticles 4-SLNs and 4-LPHNPs upon Levels of Bax, Bcl-2, Caspase-3 and p53

The tumor suppressor protein p53 leads to either growth arrest or apoptosis, acts as a transcription factor and monitors the expression of Bax and Bcl-2 genes [34]. The balance between the essential pro-apoptotic Bax and anti-apoptotic Bcl-2 proteins controls the cell fate, as Bcl-2 boosts the survival of tumor cells through inhibition of apoptosis, while Bax potentiates the crucial apoptosis of the cells. Additionally, caspases are vital mediators of apoptosis; among them, caspase-3 is involved in the induction of apoptosis through its role in apoptotic chromatin condensation, cell shrinkage and DNA fragmentation within the cells [35]. In the present work, the cells of MCF-7 were incubated with target **4** and its nanoparticles **4-SLNs** and **4-LPHNPs** at their IC_50_ concentrations of 27.29, 6.41 and 6.65 µM, respectively, for 24 h to assess the levels of Bax, Bcl-2, caspase-3 and p53. The results (Table 3) showed that targets **4**, **4-SLNs** and **4-LPHNPs** increased the levels of Bax by 4.09, 5.55 and 2.26 fold, p-53 by 3.61, 4.58 and 4.50 fold, and caspase-3 by 7.70, 11.21 and 10.61 fold, respectively, compared with the untreated MCF-7 cells. On the other hand, these targets decreased the protein Bcl-2 level by 0.45, 0.29 and 0.32 fold, respectively, in comparison with the control cells.

### 2.5. In Silico Studies

#### 2.5.1. ADME Prediction

The optimal therapeutic choice can be determined by looking at absorption, distribution, metabolism and excretion (ADME) of the substances. SwissADME, a free online tool, can facilitate the investigation [36,37]. 

The ideal drug for oral delivery is detected through the Veber and Lipinski rules. The screened pyrazolo[3,4-*c*]pyridazin **4** was found to be compliant with the prior rules (TPSA ≤ 140 Å^2^, MW ≤ 500, number of rotatable bonds ≤ 10, number of H-bond donors ≤ 5, number of hydrogen bond acceptors ≤ 10 and MLogP ≤ 4.15) (Table 4).

**Figure 7:**
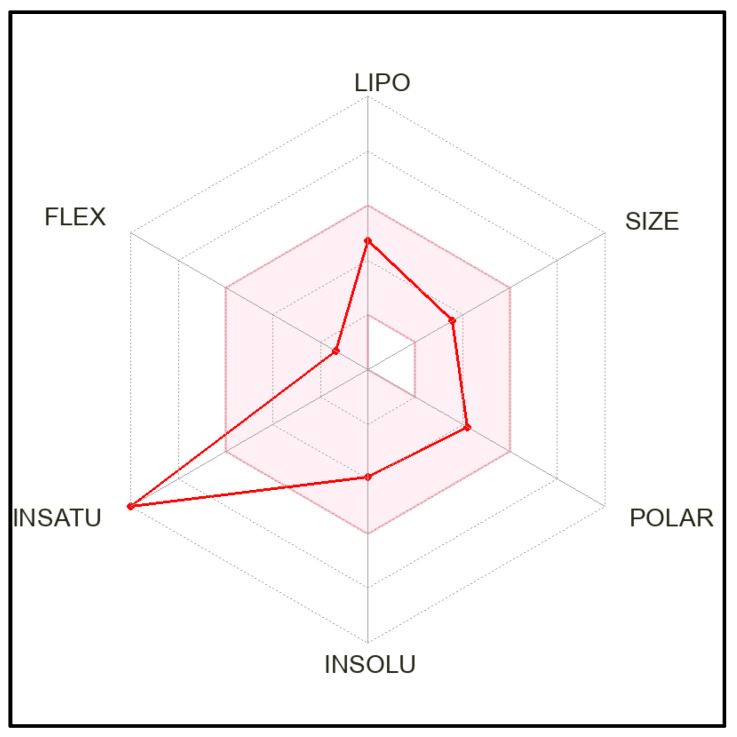
The pyrazolo-pyridazine **4** bioavailability radar map. The predicted values for the evaluated compounds are displayed as red lines, while the ideal values for each oral bioavailability item are in the pink zone.

The screened pyrazolo-pyridazine **4** was in the ideal range (pink region) on the bioavailability radar chart in regard to five important factors (Lipophilicity, size, polarity, solubility, saturation and flexibility), but it was outside the ideal saturation region, which gave a good expectation for its oral bioavailability (Figure 7).

**Figure 8:**
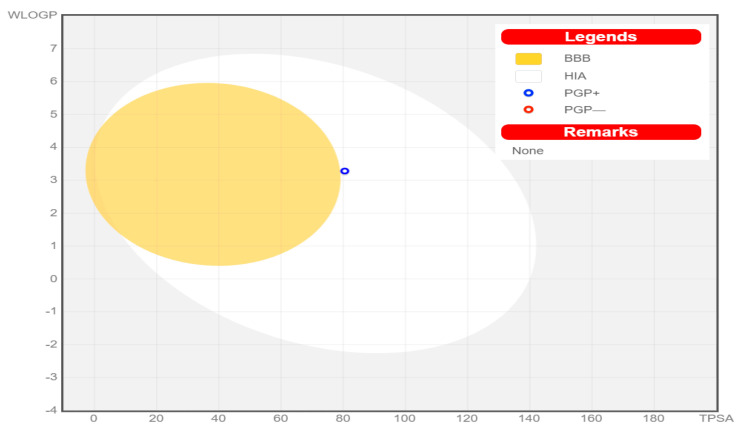
A representation of a Boiled Egg demonstrating how pyrazolo-pyridazine **4** can actively enter the gastrointestinal system but not cross the blood–brain barrier; P-glycoprotein substrate is denoted by PGP+, and P-glycoprotein non-substrate by PGP−.

Both Table S2 in the Supplementary Data and Figure 8 show the analysis of the pharmacokinetic characteristics of the potentially active pyrazolo-pyridazine **4**. It is located in the white area of the Boiled Egg chart, away from the yellow area, indicating that there was no BBB penetration and an elevated possibility of gastrointestinal absorption. It can only be used to treat peripheral infections as a result, and there is no concern that it would harm the central nervous system. This target did not appear to trigger a PAIN warning and also had a high bioavailability value of 0.55.

One of the causes of drug resistance may be the drug efflux transporter p-glycoprotein (P-gp), which is known to carry drugs outside of cells. Derivative **4** is depicted as a blue dot and was expected to be a P-glycoprotein (PGP+) substrate (Figure 8). 

#### 2.5.2. Molecular Docking

Based upon the kinase inhibitory assessment against EGFR and CDK-2/cyclin A2, a molecular docking simulation was performed to clarify the hypothetical binding modes through MOE-Dock software version 2014.0901 [38,39,40]. The PDB codes 1M17 and 3DDQ were downloaded from the protein data bank for EGFR and CDK-2/cyclinA2 kinases with their co-crystalized ligands erlotinib and roscovitine, respectively [32,41]. Firstly, re-docking of the native ligands to validate the docking process was achieved, and afforded energy scores of −10.27 and −9.55 kcal/mol at root mean square deviation (RMDS) values of 0.81 and 1.22 Å between the co-crystallized inhibitor poses and the docked poses, respectively.

**Figure 9:**
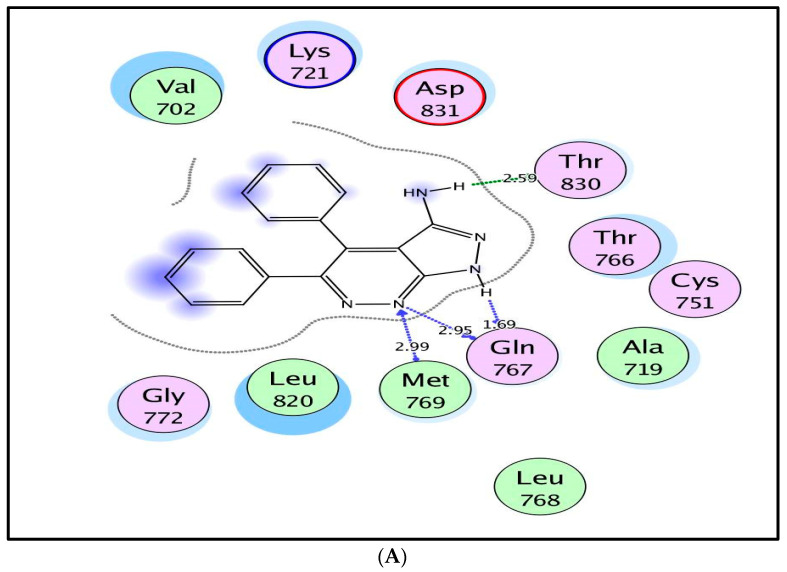

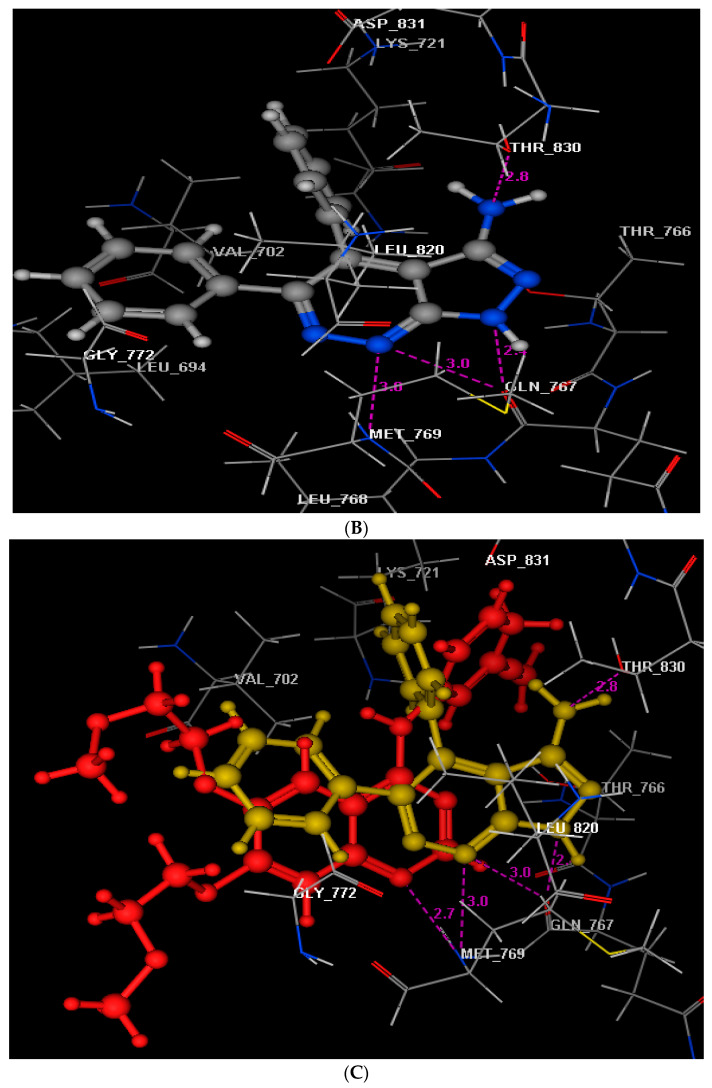
(**A**,**B**) images illustrating 2D and 3D binding modes of the target **4** within the active site of EGFR kinase (PDB code: 1M17). (**C**) Three-dimensional image of superimposition between the native ligand erlotinib (red) and the target **4** (yellow) within the active site of EGFR (PDB code: 1M17).

Concerning the active site of EGFR, target **4** docked well, giving a lower energy score (−11.35 kcal/mol) in comparison with the reference, erlotinib. As shown in Figure 9A,B, the NH at p-1 and N-7 nitrogens of pyrazolo-pyridazine moiety afforded three H-bonds with the backbones of the essential amino acids **Gln767** and **Met769** (distance: 1.69, 2.95 and 2.99 Å, respectively). Moreover, the NH_2_ group at p-3 exhibited H-bond interactions with the sidechain of **Thr830** (distance: 2.59 Å, respectively).

**Figure 10:**
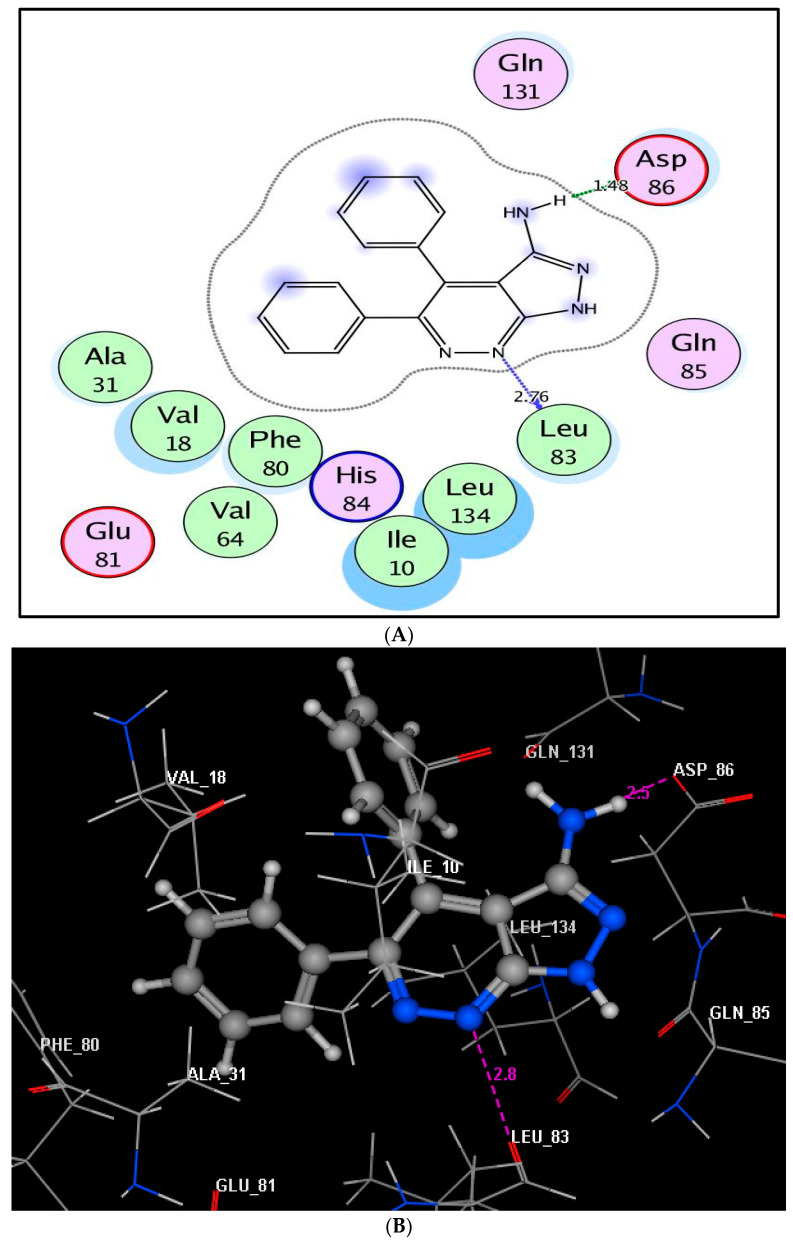

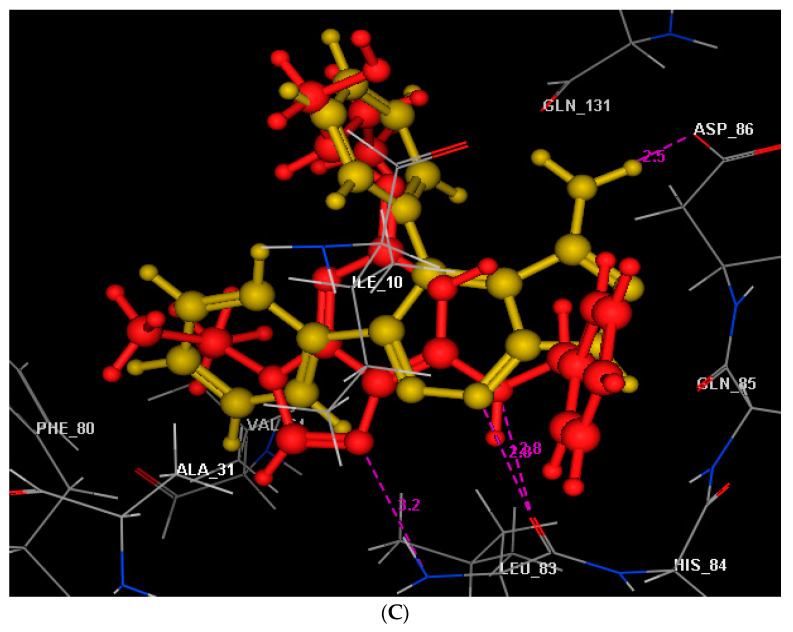
(**A**,**B**) images illustrating 2D and 3D binding modes of the target **4** within the active site of CDK-2/cyclin A kinase (PDB ID: 3DDQ). (**C**) Three-dimensional image of superimposition between the native ligand roscovitine (red) and the target **4** (yellow) within the active site of CDK-2/cyclin A kinase (PDB ID: 3DDQ).

In addition, the pyrazolo-pyridazine **4** was enclosed within the active site of CDK-2/cyclin A2 through different interactions with a promising energy score −11.27 kcal/mol. The pyrazolo-pyridazine scaffold interacted through H-bonding between N-7 with the backbone of **Leu83** in the hinge region (distance: 2.76 Å). The amino group at p-3 also displayed H-bonding with **Asp86** (distance: 1.48 Å) (Figure 10A,B).

From the docking study, it could be concluded that target **4** occupied the same active sites of EGFR and CDK-2/cyclin A2 in a similar manner with the native ligands erlotinib and roscovitine, respectively, and that was confirmed theoretically through superimposition figures (Figure 9C and Figure 10C, respectively). Additionally, the existence of the pyrazolo-pyridazine nucleus facilitates fitting within the binding sites of the screened receptors and could be the reason for the promising inhibitory activity of target **4** against both enzymes.

## 3. Materials and Methods

### 3.1. Synthesis

Compounds **1**–**3** were synthesized according to methods from the literature [21].

#### 4,5-Diphenyl-1*H*-pyrazolo[3,4-*c*]pyridazin-3-amine (**4**)

A mixture of hydrazinyl-pyridazinone (**3**) (0.01 mol) and ethyl cyanoacetate (0.01 mol) was heated under reflux in boiling dioxane (25 mL) for 4 h. The solid product formed while cooling to room temperature was filtered off and recrystallized from ethanol to give compound **4** (Yield 91%); brown crystals; m.p. 240–242 °C; IR (KBr) (ν_max_, cm^−1^): 3322, 3149, 3116 (NH, NH_2_), 3057 (CHarom), 1613 (C=N); ^1^H NMR(DMSO-*d6*) d: 4.66 (br.s, 2H, NH_2_, exchangeable), 7.23–7.43 (m, 10H, ArH), 13.06 (br.s,1H, NH, exchangeable); ^13^C NMR (DMSO-*d*_6_) δ: 106.32, 127.58, 127.85, 128.16, 129.01, 129.25, 129.87, 130.70, 130.84, 132.56, 133.86, 138.04, (14C), 148.06, 150.57, 154.98 (3C, C=N); MS (70 eV) *m*/*z* (%): 287 (M^+.^, 64), 271 (15), 259 (10), 245 (9), 230 (18), 173 (5), 117 (6), 77 (36), 57 (4); Anal. calcd. for C_17_H_13_N_5_ (287.33): C, 71.06; H, 4.56; N, 24.37. Found: C, 70.71; H,4.28; N, 24.08.

### 3.2. Crystal Structure Determination

An Agilent SuperNova Dual Atlas diffractometer using mirror monochromatedCuKα radiation was used to collect single crystal diffraction data. The structure was solved using SHELXT [42] and refined using the full-matrix least-squares methods on F^2^ with SHELXL [43]. The X-ray crystallographic data for compound **4** have been deposited in the Cambridge Crystallographic Data Center with CCDC reference number 2087298.

### 3.3. Development of Target **4** into Nano-Formulations

Materials: Eudragit L100, Suppocire CM and Poloxamer 407 were supplied from Egyptian International Pharmaceutical Industries Company (EIPICO), Cairo, Egypt.

#### 3.3.1. Formulation of Compound **4** into Solid-Lipid Nanoparticles (4-SLNs) and Lipid–Polymer Hybrid Nanoparticles (4-LPHNPs)

Nanoparticles of target **4** were formulated via the emulsification solvent evaporation technique using a previously published method [44]. Briefly, for **4-SLNs**, 100 mg suppocire were dissolved in 3 mL of dichloromethane. Compound **4** (10 mg) was also dissolved in the same amount of dichloromethane and then added to the lipid phase. An aqueous phase of Poloxamer solution was prepared (10 mL, 3% *w*/*v*). The lipid phase was added dropwise to the aqueous phase with continuous stirring at high speed for 2 h. The dichloromethane was allowed to evaporate at room temperature. The dispersion was then centrifuged at 2000 rpm for 10 min, and the supernatant was discarded. The solid precipitate was washed with distilled water and then allowed to dry at room temperature [44]. For **4-LPHNPs**, a mixture of suppocire and Eduragit L100 was used as the lipid phase and the same procedure was followed.

#### 3.3.2. Characterization of Developed Nano-Formulations (Particle Size and Zeta Potential)

The nanoparticle formulations of compound **4** were characterized for particle size (nm) and zeta potential (mV) through the dynamic light scattering technique. Particle size of the prepared formulations was measured using a Zeta-sizer 3000 PCS (Malvern Instr., Malvern, UK) equipped with a 5 mW helium–neon laser with a wavelength output of 633 nm. Measurements were made at 25C, angle 90, run time at least 180 s. Data were interpreted using the method of cumulants. The samples were diluted with double-distilled water (1:100) prior to the measurements. Zeta potential values were evaluated by using the same equipment in a single-use capillary zeta cell at 25 °C and dissolved in deionized water. The particle size and ZP values given are the averages of 3 measurements [45,46].

### 3.4. Biological Evaluation

#### 3.4.1. In Vitro Cytotoxic Screening

The in vitro antiproliferative activity of target **4** and its nanoparticles **4-SLNs** and **4-LPHNPs** was carried out against human liver HepG-2, colorectal (HCT-116) and breast (MCF-7) cancer cell lines and compared with the reference drug, doxorubicin, through the colorimetric MTT assay [27,28]. The cell lines were obtained from the Karolinska Center, Department of Oncology and Pathology, Karolinska Institute and Hospital, Stockholm, Sweden. All experiments were performed in triplicate and were repeated on three different days. All values were recorded as mean ± SD. The IC_50_s were calculated using the SPSS probit analysis software program (SPSS Inc., Chicago, IL, USA). More details are cited in the Supplementary Data.

#### 3.4.2. In Vitro EGFR and CDK-2 Inhibitory Assessment

The assay of EGFR and CDK-2/cyclin A2 was achieved for target **4** and its nanoparticles **4-SLNs** and **4-LPHNPs** following the reported procedure and cited in the Supplementary Data [29,30,31,32,33].

#### 3.4.3. Effect of Target **4** and Its Nanoparticles **4-SLNs** and **4-LPHNPs** upon Levels of Bax, Bcl-2, Caspase-3 and p53

The levels of the apoptotic marker Bax, the anti-apoptotic marker Bcl-2 and tumor suppressor gene p53 were estimated using BIO RAD iScript TM One-Step RT-PCR kit with SYBR^®^ Green. The activity of caspases-3 was assessed using DRG Caspase-3 (human) ELISA (EIA-4860) kit (DRG International Inc., Springfield, NJ, USA). Manufacturer’s instructions were followed when using the kits [34,35].

### 3.5. Molecular Docking Study

The co-crystallized structures of EGFR and CDK-2/cyclin A2 kinases (PDB codes: 1M17 and 3DDQ) [32,41] complexed with erlotinib and roscovitine, respectively, were downloaded from the RCSB Protein Data Bank. The docking simulation was achieved using Molecular Operating Environment (MOE-Dock) software version 2014.0901 [38,39,40], following the reported procedure and cited in details in the Supplementary Data.

## 4. Conclusions

After validating the chemical structure of the previously synthesized 4,5-diphenyl-1*H*-pyrazolo[3,4-*c*]pyridazin-3-amine (**4**) with single crystal X-ray analysis, nanoparticles **4-SLNs** and **4-LPHNPs** were prepared. In comparison to the reference drug doxorubicin and the original target **4**, these nanoparticles demonstrated promising cytotoxic efficacy against the cancer cell lines HepG-2, HCT-116 and MCF-7. Additionally, inhibitory evaluation against EGFR and CDK-2/cyclin A2 demonstrated enhanced and preferable effects for the nanoparticles compared to the precursor **4** and the standards. The levels of Bax, p53 and caspase-3 were upregulated while the level of Bcl-2 was downregulated when the effect of **4-SLNs** and **4-LPHNPs** upon cancer biomarkers was established. According to the docking simulation, the pyrazolo[3,4-*c*]pyridazin-3-amine scaffold has the ability to be accommodated by and bound to the EGFR and CDK-2 receptors well via various hydrophilic interactions. Using ADME analysis, the pharmacokinetic and physicochemical characteristics of target **4** were also evaluated, and the results suggested that it has good drug-like characteristics.

## Figures and Tables

**Table 1:** In vitro cytotoxic activity of targets **4**, **4-SLNs** and **4-LPHNPs** against HepG-2, HCT-116 and MCF-7 cell lines.

Compound No.	IC_50_ (Mean ± SEM) (μM)		
	HepG-2	HCT-116	MCF-7
**4**	17.30 ± 1.42	18.38 ± 1.4	27.29 ± 2.1
**4-SLNs**	7.56 ± 0.58	4.80 ± 0.3	6.41 ± 0.4
**4-LPHNPs**	7.85 ± 0.5	5.24 ± 0.4	6.65 ± 0.5
**Doxorubicin**	6.18 ± 0.3	5.23 ± 0.3	4.17 ± 0.2

IC_50_: Concentration of the compound required to inhibit the cell viability by 50%, SEM: Standard error mean; each value is the mean of three independent determinations.

**Table 2:** The inhibitory assessment of target **4** and its nanoparticles **4-SLNs** and **4-LPHNPs**, in comparison with erlotinib and roscovitine, against EGFR and CDK-2/cyclin A2, respectively.

Compound No.	IC_50_ (Mean ± SEM) (µM)	
	EGFR	CDK-2/Cyclin A2
**Erlotinib**	0.126 ± 0.10	-
**Roscovitine**	-	0.32 ± 0.05
**4**	0.391 ± 0.17	0.55 ± 0.20
**4-SLNs**	0.088 ± 0.22	0.18± 0.15
**4-LPHNPs**	0.096 ± 0.50	0.22± 0.04

IC_50_: Compound concentration necessary to inhibit the enzyme activity by 50%, SEM: Standard error mean; each value is the mean of three values.

**Table 3:** Effect of targets **4**, **4-SLNs** and **4-LPHNPs** on the levels of Bax, Bcl-2, p53 and caspase-3.

Compd.	Bax	Bcl-2	P53	Casp-3
	Conc. Pg/mL	Conc. ng/mL	Conc. Pg/mL	Conc. Pg/mL
**4/MCF-7**	166.20 ± 0.04	2.40 ± 0.30	590.22 ± 0.60	14.25 ± 0.20
**4-SLNs/MCF-7**	225.40 ± 0.01	1.55 ± 0.20	748.10 ± 0.14	20.73 ± 0.11
**4-LPHNPs/MCF-7**	213.50 ± 0.12	1.68 ± 0.15	735.50 ± 0.22	19.62 ± 0.35
**Cont./MCF-7**	40.60 ± 0.05	5.30 ± 0.10	163.41 ± 0.22	1.85 ± 0.35

**Table 4:** Anticipated physicochemical features of the pyrazolo-pyridazine **4**.

Compd.	MW ^a^	TPSA (Å²) ^b^	nRB ^c^	nHBA ^d^	nHBD ^e^	MLogP ^f^	Violations ^g^
**4**	287.32	80.48	2	3	2	3.07	0

^a^ Molecular Weight; ^b^ Topological Polar Surface Area; ^c^ Number of Rotatable Bonds; ^d^ Number of Hydrogen Bond Acceptors; ^e^ Number of Hydrogen Bond Donors; ^f^ Calculated Lipophilicity (MLog Po/w); ^g^ Violations from Lipinski and Veber Rules.

## Data Availability

Not applicable.

## Supplementary Materials

The following supporting information can be downloaded at: https://www.mdpi.com/article/10.3390/molecules28217252/s1, include spectral and biological data of synthesized compound. Table S1. Selected geometry (Å, °] for **4**. Table S2. Anticipated Pharmacokinetic features of the potent pyrazolines **4**. IR, Mass, ^1^H-NMR and ^13^ C-NMR for compound **4**.
