# Supplementary material for: Nanoparticles of a Pyrazolo-Pyridazine Derivative as Potential EGFR and CDK-2 Inhibitors: Design, Structure Determination, Anticancer Evaluation and In Silico Studies
Source: Molecules. 2023 Oct 25;28(21):7252. doi: 10.3390/molecules28217252 (PMC10648062; doi:10.3390/molecules28217252)
Supplement: Supplementary file 1 [file molecules-28-07252-s001.zip › molecules-2586591-supplementary.pdf]

Supplementary data

Nanoparticles of a Pyrazolo-Pyridazine Derivative as Potential EGFR and CDK-2 Inhibitors: Design, Structure Determination, Anticancer Evaluation and In Silico Studies

Heba E. Hashem [1], Abd El-Galil E. Amr [2,*], Abdulrahman A. Almehizia [3], Ahmed M. Naglah [3], Benson M. Kariuki [4], Heba A. Eassa [5,6] and Eman S. Nossier [7,8]

[1] Department of Chemistry, Faculty of Women, Ain Shams University, Cairo 11757, Egypt
[2] Applied Organic Chemistry Department, National Research Center, Cairo 12622, Egypt
[3] Department of Pharmaceutical Chemistry, College of Pharmacy, King Saud University, P.O. Box 2457, Riyadh 11451, Saudi Arabia
[4] School of Chemistry, Cardiff University, Main Building, Park Place, Cardiff CF10 3AT, UK;
[5] Department of Pharmaceutics and Pharmaceutical Technology, Faculty of Pharmacy (Girls), Al-Azhar University, Cairo 11754, Egypt
[6] Department of Pharmaceutical Sciences, School of Pharmacy and Physician Assistant Studies, University of Saint Joseph, West Hartford, CT 06117, USA
[7] Department of Pharmaceutical Medicinal Chemistry and Drug Design, Faculty of Pharmacy (Girls), Al-Azhar University, Cairo 11754, Egypt
[8] The National Committee of Drugs, Academy of Scientific Research and Technology, Cairo 11516, Egypt

* Correspondence: aeamr1963@yahoo.com.

1. Material and method

1.1. Synthesis

General method

All melting points are uncorrected and were measured on a Gallenkamp electric melting point apparatus. The IR spectra were recorded using potassium bromide disks on an FTIR Thermo Electron Nicolet 7600 (USA) IR spectrometer at the Central Laboratory of Faculty of Science, Ain Shams University. ^{1}HNMR and ^{13}NMR spectra were run at 300MHz on a GEMINI 300 BB NMR spectrometer using tetramethylsilane as internal standard in deuterated dimethyl sulfoxide (DMSO-d_6) at Faculty of Pharmacy, Ain Shams University. The mass spectra were recorded on Shimadzu GCMSQP 1000EX mass spectrometer operating at 70 eV and the elemental analyses at the Micro Analytical Center of Al-Azhar University. TLC was performed on Merck Kiesel gel 60 F254 aluminumbacked plates. The spots were detected by UV irradiation at 254–365 nm.

1.2. Crystal Structure Determination

An Agilent SuperNova Dual Atlas diffractometer using mirror monochromated CuKα radiation was used to collect single crystal diffraction data. The structure of **4** was solved using SHELXT and refined by full-matrix least-squares methods on F^2 with SHELXL [41,42]. $C_{17}H_{13}N_5.H_2O$, FW =305.34, T = 296(2) K, λ = 1.54184 Å, monoclinic, $P2_1/c$, a = 10.2933(3) Å, b = 8.3038(2) Å, c = 18.4623(6) Å, β =103.913(3)°, V = 1531.74(8) $Å^3$, Z = 4, calculated density = 1.324 Mg/m^3, absorption coefficient = 0.707 mm^{-1}, F(000) = 640, crystal size = 0.340 x 0.226 x 0.175 mm^3, reflections collected = 13619, independent reflections = 3211, R(int) = 0.0218, parameters = 224, goodness-of-fit on F^2 = 1.075, R1 = 0.0403, wR2 = 0.1059 for ($I>2\sigma(I)$), R1 = 0.0460, wR2 = 0.1132 for all data, largest difference peak and hole = 0.186 and 0.205 $e.Å^{-3}$. The X-ray crystallographic data for compound **4** have been deposited in the Cambridge Crystallographic Data Center with CCDC reference number 2087298.

1.1. Biological activity

1.1.1. *In vitro* cytotoxic assay (MTT)

Cell culture conditions

The cells of human liver carcinoma (HepG-2), colorectal carcinoma (HCT-116) and breast adenocarcinoma (MCF-7) were obtained from Karolinska Center, Department of Oncology and Pathology, Karolinska Institute and Hospital, Stockholm, Sweden. All cells were maintained in a DMEM medium, which was supplemented with 10% of heat-inactivated fetal bovine serum (FBS), 100U/ml of each of penicillin and streptomycin. The cells were grown at 37°C in a humidified atmosphere of 5% CO_2.

MTT antiproliferative assay

The antiproliferative activities on the HepG-2, HCT-116 and MCF-7 human cancer cell lines were estimated, employing the 3-[4,5-dimethyl-2-thiazolyl)-2,5-diphenyl-2H-tetrazolium bromide (MTT) assay, which was grounded on the reduction of the tetrazolium salt by the mitochondrial dehydrogenases in viable cells. The cells were dispensed in a 96 well sterile microplate (3 x 10^4 cells/well), followed by their incubation at 37°C with a series of different concentrations of 10 μl of each compound or Doxorubicin® (positive control, in DMSO) for 48 h in a serum free medium prior to the MTT assay. Subsequently, the media were carefully removed, 40 μL of MTT (2.5 mg/mL) were added to each well, and then incubated for an additional 4 h. The purple formazan dye crystals were solubilized by the addition of 200 μL of

DMSO. The absorbance was measured at 570 nm applying a SpectraMax® Paradigm® Multi-Mode microplate reader. The relative cell viability was expressed as the mean percentage of viable cells relative to the untreated control cells. All experiments were conducted in triplicate and were repeated on three different days. All the values were represented as mean ± SD. The IC_{50}s were determined by the SPSS probit analysis software program (SPSS Inc., Chicago, IL).

1.1.2. *In vitro* inhibition assay of EGFR and CDK-2 activities

The target **4** and its nanoparticles **4-SLNs** and **4-LPHNPs** were further examined for their inhibitory activities against EGFR and CDK-2/cyclin A2.

EGFR assay: The master mixture (6 µL 5X Kinase Buffer + 1 µL ATP (500 µM) + 1 µL 50 X PTK substrate + 17 µL water) was prepared then, 25 µL to every well was added. 5 µL of Inhibitor solution of each well labeled as “Test Inhibitor” was added. However, for the “Positive Control" and “Blank”, 5 µL of the same solution without inhibitor (Inhibitor buffer) was added. 3 mL of 1X Kinase Buffer by mixing 600 µL of 5X Kinase Buffer with 2400 µL water was prepared. So, 3 mL of 1X Kinase Buffer became sufficient for 100 reactions. To the wells designated as "Blank", 20 µl of 1X Kinase Buffer was added. EGFR enzyme on ice was thawed. Upon first thaw, briefly the tube containing enzyme was spun to recover full content of the tube. The amount of EGFR required for the assay and dilute enzyme to 1 ng/µL with 1X Kinase Buffer was calculated. Moreover, the remaining undiluted enzyme in aliquots was stored at -80°C. The reaction was initiated by adding 20 µL of diluted EGFR enzyme to the wells designated “Positive Control” and "Test Inhibitor Control", after that it was incubated at 30°C for 40 minutes. After the 40 minutes reaction, 50 µL of Kinase-Glo Max reagent was added to each well and the plate was covered with aluminum foil and incubated at room temperature for 15 min. Luminescence was measured using the microplate reader.

Assay of CDK-2/cyclin A2 was achieved through ELISA using an affinity tag labeled capture antibody. Adding the samples and the standard to the wells has been done after that addition of the antibody mix. After the incubation period, the unrestrained substance has been discarded and the wells have been washed. The added TMB (3,3',5,5'-tetramethylbenzidine)

substrate was prompted by Horseradish peroxidase (HRP) and blue coloration was appeared. This reaction was Stopped through addition of stop solution to complete changing in color from blue to yellow. The created signals were equivalent to the quantity of bound analyte and Robonik P2000 ELISA reader was used to record the intensity at certain wavelength (450 nm). The concentrations of the screened compounds have been calculated through the plotted curve.

1.2. Molecular docking study on EGFR and CDK-2

The 2D structure of the target **4** and its nanoparticles **4-SLNs** and **4-LPHNPs** were drawn through chem. Draw. The protonated 3D was employed using standard bond lengths and angles, using Molecular Operating Environment (MOE-Dock) software version 2014.0901. Then, the geometry optimization and energy minimization were applied to get the Conf Search module in MOE, followed by saving of the moe file for upcoming docking process. The co-crystallized structures of EGFR and CDK-2/cyclin A2 kinases with their ligands erlotinib and roscovitine were downloaded (PDB codes: 1M17 and 3DDQ, respectively) from protein data bank. All minimizations were performed using MOE until an RMSD gradient of 0.05 kcal·mol^{-1}Å^{-1} with MMFF94x force field and the partial charges were automatically calculated. Preparation of the enzyme structures was done for molecular docking using Protonate 3D protocol with the default options in MOE. London dG scoring function and Triangle Matcher placement method were used in the docking protocol. At the first, validation of the docking processes were established by docking of the native ligands, followed by docking of the target **4** and its nanoparticles **4-SLNs** and **4-LPHNPs** within the ATP-binding sites after elimination of the co-crystallized ligands.

Table S1. Selected geometry (Å, °] for **4**.

C(1)-N(2)	1.3192(16)	C(3)-N(3)	1.3455(16)
C(1)-N(1)	1.3683(17)	C(4)-C(5)	1.4110(16)
C(1)-C(2)	1.4368(16)	C(5)-N(5)	1.3496(15)
C(2)-C(4)	1.3869(16)	N(2)-N(3)	1.3845(16)
C(2)-C(3)	1.4131(16)	N(4)-N(5)	1.3341(15)
C(3)-N(4)	1.3383(16)	N(4)-C(3)-C(2)	125.69(11)
N(2)-C(1)-N(1)	121.77(12)	N(3)-C(3)-C(2)	107.75(11)
N(2)-C(1)-C(2)	110.31(11)	C(2)-C(4)-C(5)	114.89(10)
N(1)-C(1)-C(2)	127.88(11)	N(5)-C(5)-C(4)	124.01(11)
C(4)-C(2)-C(3)	117.52(11)	C(1)-N(2)-N(3)	107.25(10)
C(4)-C(2)-C(1)	138.27(11)	C(3)-N(3)-N(2)	110.54(10)
C(3)-C(2)-C(1)	104.14(10)	N(5)-N(4)-C(3)	116.25(10)
N(4)-C(3)-N(3)	126.54(11)	N(4)-N(5)-C(5)	121.61(10)

	**D…A**	**D-H…A**

N(3)-H(3)...O(1)	2.7939(15)	149.4
N(1)-H(2N)...N(5)#1	3.3038(16)	166.4(15)
O(1)-H(3N)...N(4)#2	2.9851(16)	176(3)
O(1)-H(4N)...N(2)#3	2.9899(16)	169(2)

#1 x,y+1,z #2 -x+2,y+1/2,-z+3/2 #3 -x+2,y-1/2,-z+3/2

Table S2. Anticipated Pharmacokinetic features of the potent pyrazolines **4**.

Comp. No.	GIT absorption	BBB permeability	P-gp substrate	Bioavailability score	PAINS alert
**4**	High	NO	Yes	0.55	0

Spectroscopic analyses of 4,5-diphenyl-1*H*-pyrazolo[3,4-*c*]pyridazin-3-amine (4):

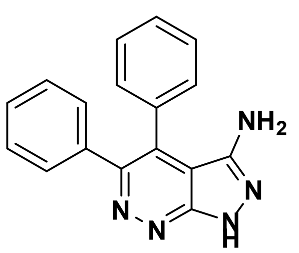

4

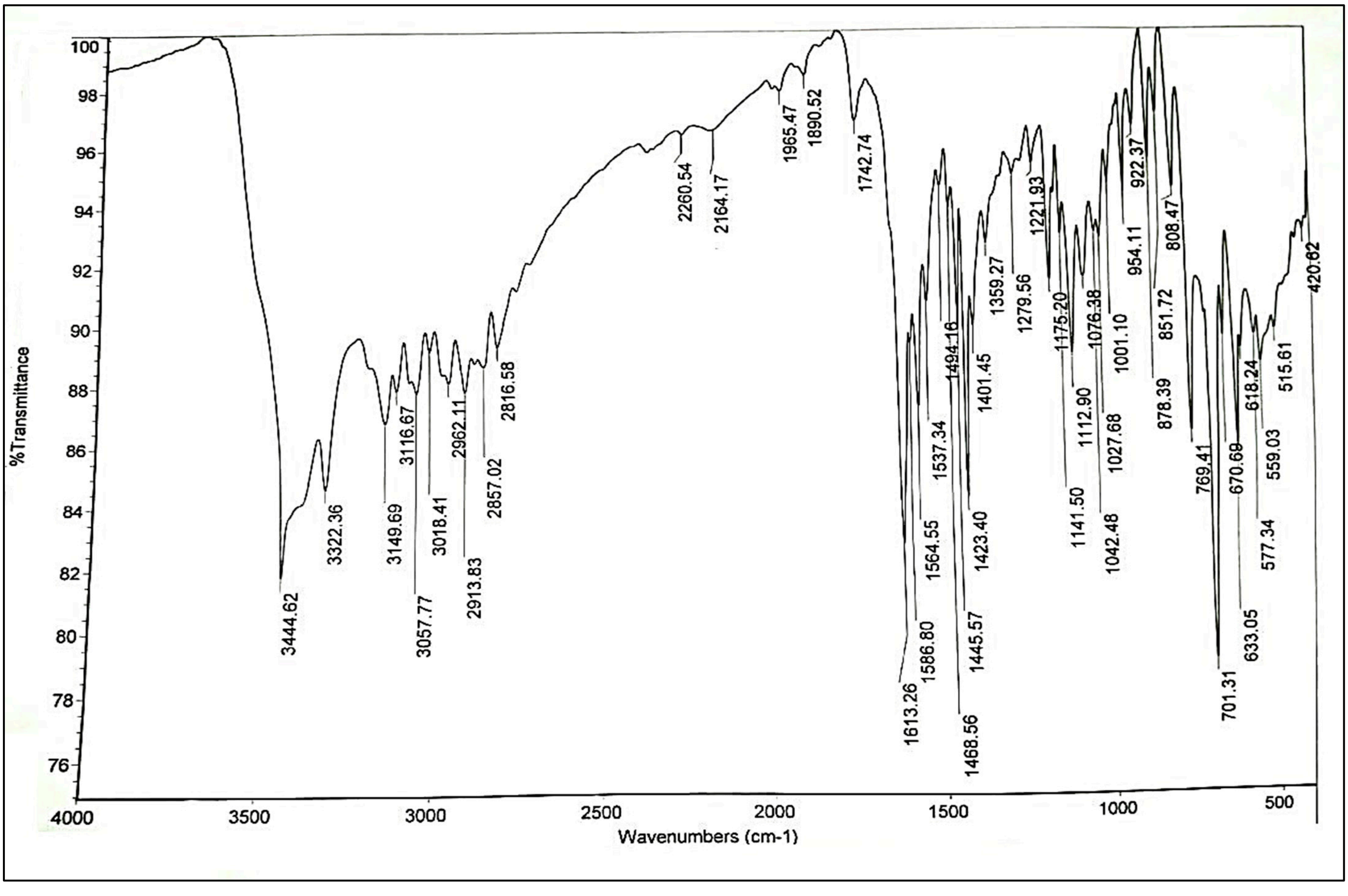

IR spectrum of 4,5-diphenyl-1*H*-pyrazolo[3,4-*c*]pyridazin-3-amine (**4**)

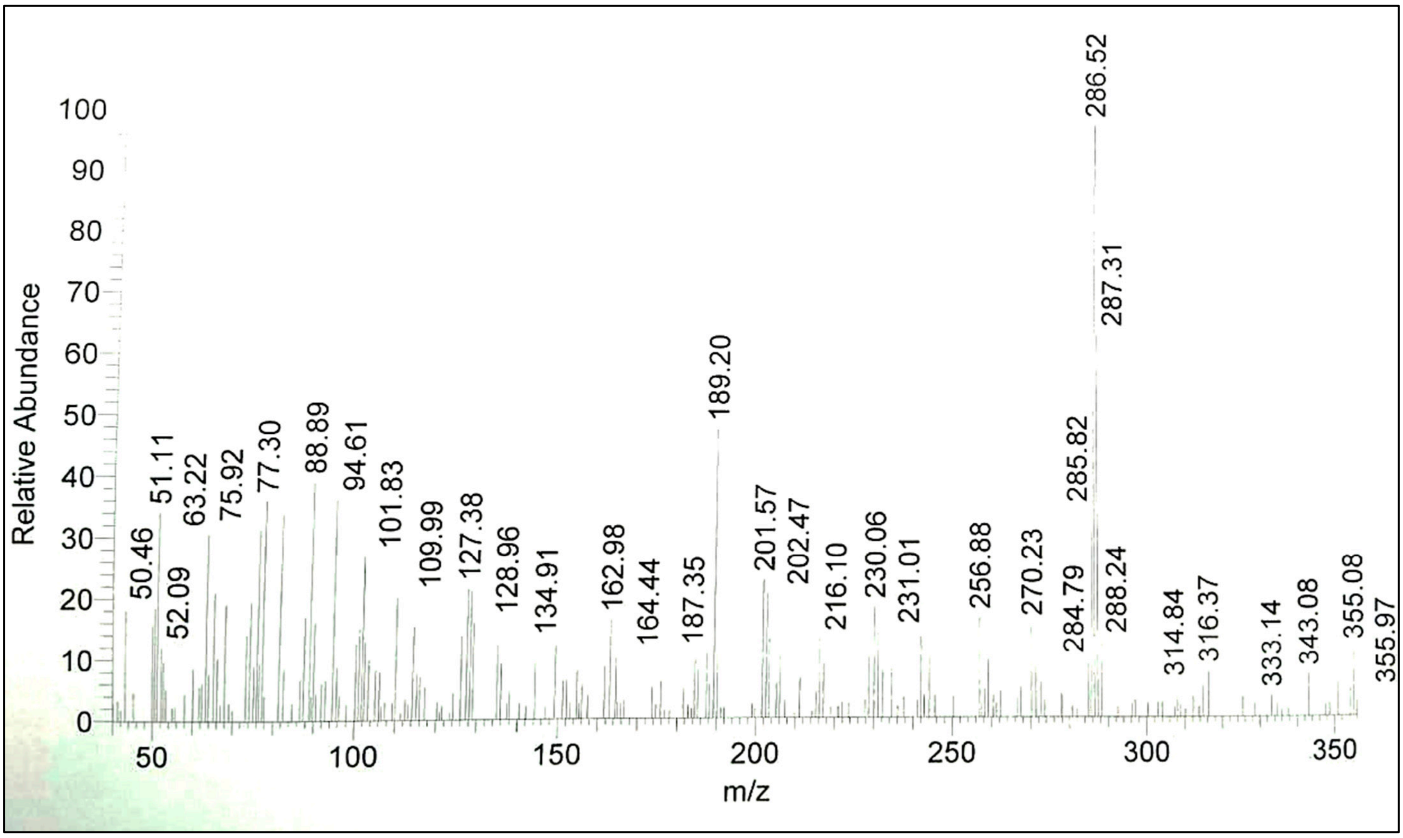

Mass spectrum of 4,5-diphenyl-1*H*-pyrazolo[3,4-*c*]pyridazin-3-amine (**4**)

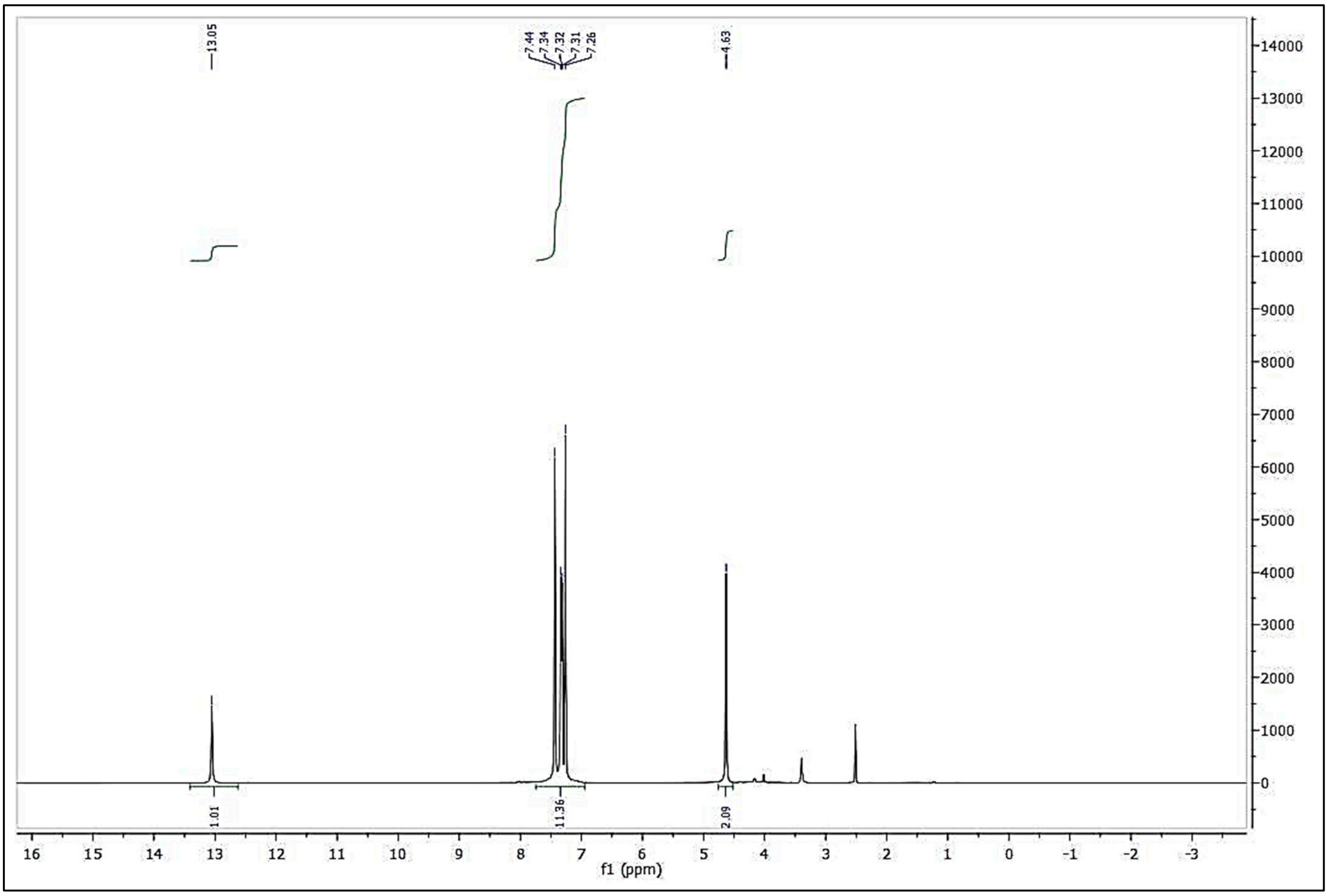

^{1}HNMR spectrum of 4,5-diphenyl-1*H*-pyrazolo[3,4-*c*]pyridazin-3-amine (**4**)

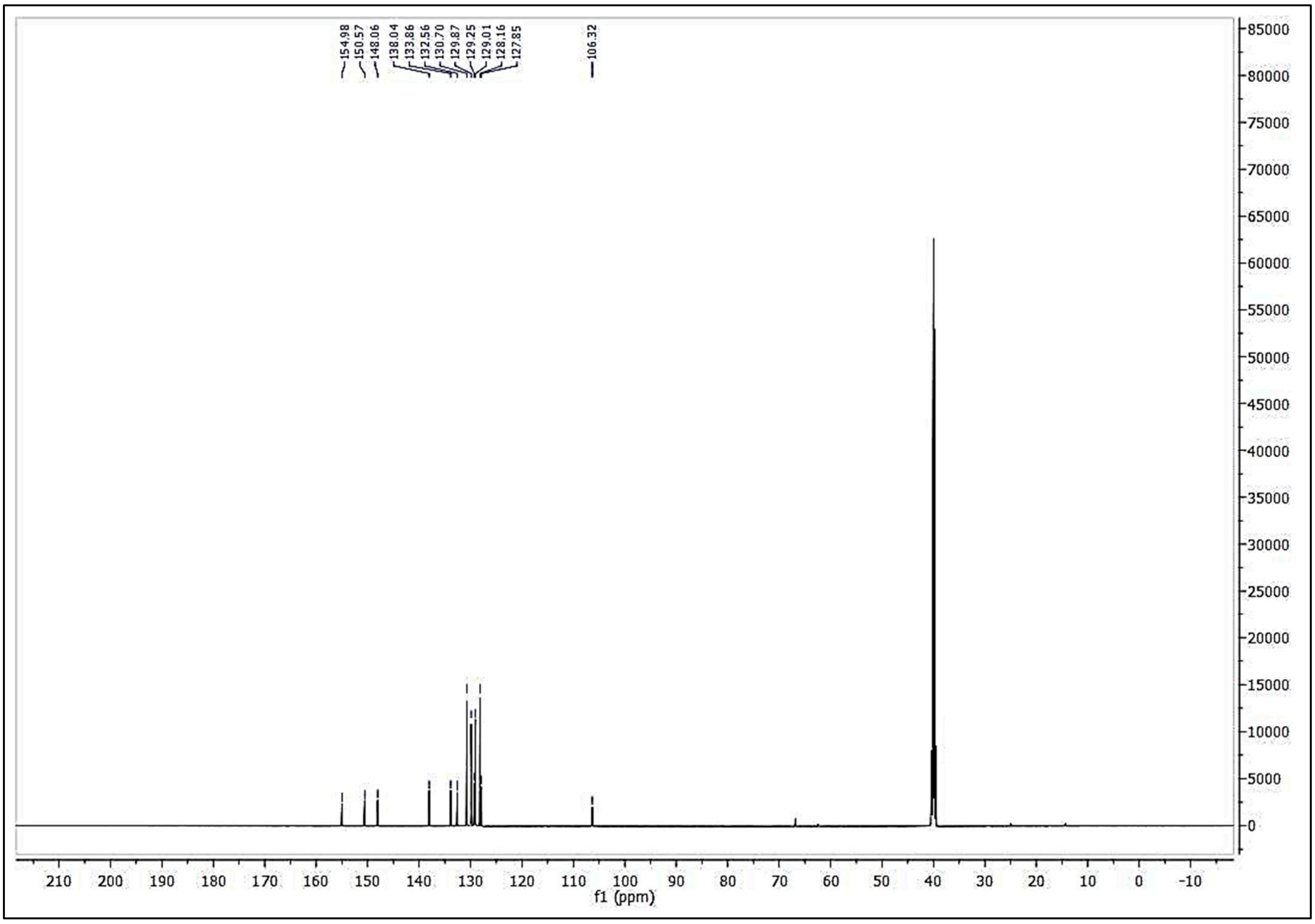

^{13}CNMR spectrum of 4,5-diphenyl-1*H*-pyrazolo[3,4-*c*]pyridazin-3-amine (**4**)
